# A comparison of the chemical reactivity of naringenin calculated with the M06 family of density functionals

**DOI:** 10.1186/1752-153X-7-155

**Published:** 2013-09-16

**Authors:** Daniel Glossman-Mitnik

**Affiliations:** 1 NANOCOSMOS Virtual Lab, CIMAV, Miguel de Cervantes 120, Complejo Industrial Chihuahua, Chihuahua, Chih 31109, Mexico

**Keywords:** Naringenin, DFT, M06 density functionals, Conceptual DFT, Chemical reactivity

## Abstract

**Background:**

Chemicals generically referred to as flavonoids belong to the group of phenolic compounds and constitute an important group of secondary metabolites due to their applications as well as their biochemical properties. Flavonoids, which share a common benzo- *γ*-pyrone structure, constitute a kind of compound which are highly ubiquitous in the plant kingdom.

**Findings:**

The M06 family of density functionals has been assessed for the calculation of the molecular structure and properties of the Naringenin flavonoid. The chemical reactivity descriptors have been calculated through Conceptual DFT. The active sites for nucleophilic and electrophilic attacks have been chosen by relating them to the Fukui function indices and the dual descriptor *f*^(2)^(**r**). A comparison between the descriptors calculated through vertical energy values and those arising from the Koopmans’ theorem approximation have been performed in order to check for the validity of the last procedure.

**Conclusions:**

The M06 family of density functionals (M06, M06L, M06-2X and M06-HF) used in the present work leads to the same qualitatively and quantitatively similar description of the chemistry and reactivity of the Naringenin molecule, yielding reasonable results. However, for the case of the M06-2X and M06-HF density functionals, which include a large portion of HF exchange, the calculations considering the validity of the Koopmans’ theorem lead to negative electron affinities.

## Findings

### Introduction

Chemicals generically referred to as flavonoids belong to the group of phenolic compounds and constitute an important group of secondary metabolites due to their applications as well as their biochemical properties. Flavonoids, which share a common benzo- *γ*-pyrone structure, constitute a kind of compound which are highly ubiquitous in the plant kingdom. Over 4, 000 different naturally occurring flavonoids have been discovered, and only in the case of flavones, a specific type of flavonoids, over 36, 000 different chemical structures are possible. Flavonoids are present in a wide variety of edible plant sources, such as fruits, vegetables, nuts, seeds, grains, tea and wine [1].

The knowledge of reactivity on a molecule is an essential concept; it is of a crucial interest because it allows to understand interactions that are operating during a reaction mechanism. In particular electrostatic interactions have been successfully explained by the use of the molecular electrostatic potential [2,3].

On the other hand, there is no a unique tool to quantify and rationalize covalent interactions, however since 2005 a descriptor of local reactivity whose name is simply dual descriptor [4,5], has allowed to rationalize reaction mechanisms in terms of overlapping nucleophilic regions with electrophilic regions in order to get a maximum stabilization thus leading to final products or intermediates; all those favorable nucleophilic–electrophilic interactions have been explained as a manifestation of the Principle of Maximum Hardness [6] in addition, chemical reactions have been understood in terms of the The Hard and Soft Acids and Bases Principle [7-10], principle that has been used even with the aim of replacing the use of the Molecular Orbital Theory to understand the whole Chemistry [11]. In fact the present work is a good chance to test the capability of the most recent reactivity descriptors coming from the Conceptual DFT [12-15], therefore the framework of this conceptual theory will be presented in the next section.

**Figure 1:**
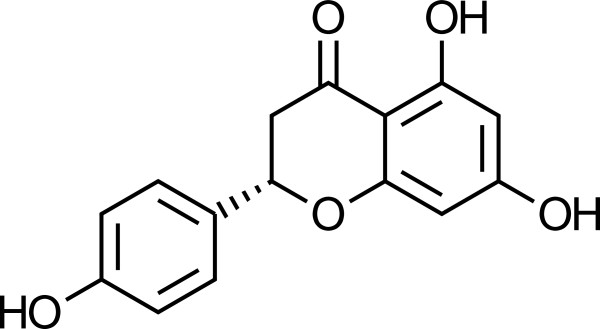
**A sketch of the molecular structure of the naringenin flavonoid.** This figure shows a sketch of the molecular structure of the naringenin flavonoid.

**Figure 2:**
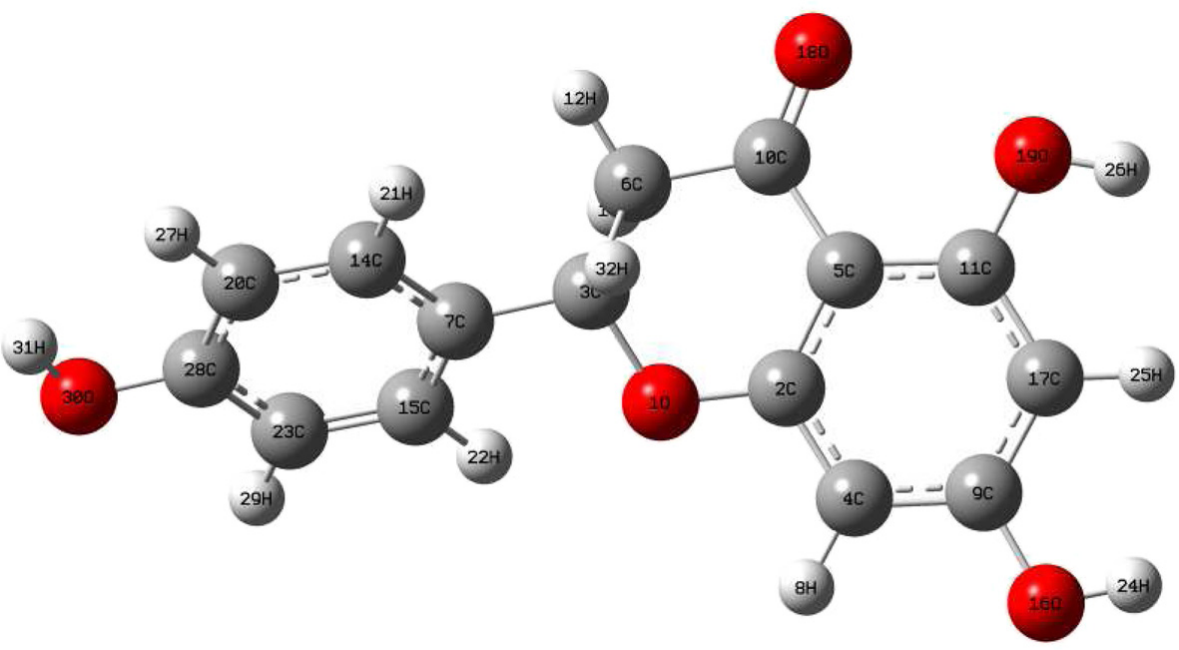
**Optimized molecular structure of the naringenin flavonoid.** This figure displays the optimized molecular structure of the naringenin flavonoid, showing the atoms numbers and symbols.

Naringenin (C15H12O5; mol. wt. 272.3; IUPAC (2S)-5,7-Dihydroxy-2-(p-hydroxyphenyl)-4-chromanone, is a plant flavonoid that is extracted from citrus fruit. Its properties are estimated as follows: Polarizability = 27.3945 *Å*^3^, Molar Refractivity = 70.619 cm^3^/mol, Polar Surface Area = 86.99 *Å*^2^, vdW volume = 234.6628 *Å*^3^, logP = 3.398, and Complexity = 476.8368. It must be kept in mind that the objective of this work is not to perform an evaluation of the antioxidant properties of Naringenin, but to do a comparative study of the performance of the M06 family of density functionals for the description of the chemical reactivity of this prototypical flavonoid whose molecular structure is shown in Figures 1 and 2.

### Theory and computational details

Morell et al. [5,9,11,16-19] have proposed a local reactivity descriptor (LRD) which is called the dual descriptor (DD) *f*^(2)^(**r**) ≡ Δ*f*(**r**). In spite of having been discovered several years ago, a solid physical interpretation was not provided in such a moment. [20]. They used the notation Δ*f*(**r**), but currently it has been replaced by the modern notation *f*^(2)^(**r**) in order to highlight that this is a Fukui function of second order. Its physical meaning is to reveal nucleophilic and electrophilic sites on a molecular system at the same time. Mathematically it is defined in terms of the derivative of the Fukui function, *f*(**r**) [14], with respect to the number of electrons, *N*. Through a Maxwell relation, this LRD may be interpreted as the variation of *η* (the molecular hardness which measures the resistance to charge transfer [21]) with respect to *υ*(**r**), the external potential. The definition of *f*^(2)^(**r**) is shown as indicated by Morell et al. [5,9]:

(1) $$f^{\left(2\right)}(r)={\left(\frac{\partial f(r)}{\partial N}\right)}_{\upsilon (r)}={\left[\frac{\delta \eta }{\delta \upsilon (r)}\right]}_{N}.$$

As mentioned above, DD allows one to obtain simultaneously the preferably sites for nucleophilic attacks (*f*^(2)^(**r**) > 0) and the preferably sites for electrophilic attacks (*f*^(2)^(**r**) < 0) into the system at point **r**. DD has demonstrated to be a robust tool to predict specific sites of nucleophilic and electrophilic attacks in a much more efficient way than the Fukui function by itself because dual descriptor is able to distinguish those sites of true nucleophilic and electrophilic behavior, in consequence some works have been published with the aim of remarking the powerfulness of *f*^(2)^(**r**) and all those LRDs depending on DD [5,9,11,16-19].

The general working equation to obtain DD is given by the difference between nucleophilic and electrophilic Fukui function [11]. A well–known first level of approximation implies the use of finite difference method where to the sum of electronic densities of the system with one more electron and one less electron is subtracted by the double of the total electronic density of the original system. Since this level of approximation implies a time–demanding computing, a second level of approximation has been used for some years where the densities of FMOs provide an easier–to–compute working equation:

(2) $$\begin{array}{l} f^{\left(2\right)}(r)=f^{+}(r)-f^{-}(r)≃\rho_{{}_{L}}(r)-\rho_{{}_{H}}(r), \end{array}$$

where densities of LUMO and HOMO are represented by $\rho_{{}_{L}}(r)$ and $\rho_{{}_{H}}(r)$, respectively.

Hence, when an interaction between two species is well described through the use of this LRD, it is said the reaction is controlled by frontier molecular orbitals (or frontier–controlled) under the assumption that remaining molecular orbitals do not participate during the reaction.

The dual descriptor can also be condensed through an appropriate integration within the *k*^th^–atomic domain Ω_*k*_:

(3) $$\int_{\Omega_{k}}f^{\left(2\right)}(r)dr=f_{k}^{\left(2\right)}.$$

When $f_{k}^{\left(2\right)}>0$ the process is driven by a nucleophilic attack on atom *k* and then that atom acts an electrophilic species; conversely, when $f_{k}^{\left(2\right)}<0$ the process is driven by an electrophilic attack over atom *k* and therefore atom *k* acts as a nucleophilic species.

### Settings and computational methods

All computational studies were performed with the Gaussian 09 [22] series of programs with density functional methods as implemented in the computational package. The equilibrium geometries of the molecules were determined by means of the gradient technique. The force constants and vibrational frequencies were determined by computing analytical frequencies on the stationary points obtained after the optimization to check if there were true minima. The basis set used in this work was MIDIY, which is the same basis set as MIDI! with a polarization function added to the hydrogen atoms. The MIDI! basis is a small double-zeta basis with polarization functions on N-F, Si-Cl, Br, and I [23-28].

For the calculation of the molecular structure and properties of the studied system, we have chosen the hybrid meta-GGA density functionals M06, M06L, M06-2X and M06HF [29], which consistently provide satisfactory results for several structural and thermodynamic properties [29-31]. All the calculations were performed in the presence of water as a solvent, by doing IEFPCM computations according to the SMD solvation model [32].

Within the conceptual framework of DFT [14,21], the chemical potential *μ*, which measures the escaping tendency of electron from equilibrium, is defined as:

(4) $$\mu ={\left(\frac{\partial E}{\partial N}\right)}_{v(\vec{r})}=-\chi$$

where *χ* is the electronegativity.

The global hardness *η* can be seen as the resistance to charge transfer:

(5) $$\eta =\frac{1}{2}{\left(\frac{\partial^{2}E}{\partial N^{2}}\right)}_{v(\vec{r})}$$

Using a finite difference approximation and Koopmans’ theorem [25-28], the above expressions can be written as:

(6) $$\mu \approx -\frac{1}{2}(I+A)\approx \frac{1}{2}(\varepsilon_{L}+\varepsilon_{H})$$

(7) $$\eta \approx \frac{1}{2}(I-A)\approx \frac{1}{2}(\varepsilon_{L}-\varepsilon_{H})$$

where *ε*_*H*_ and *ε*_*L*_ are the energies of the highest occupied and the lowest unoccupied molecular orbitals, HOMO and LUMO, respectively. However, within the context of density functional theory, the above inequalities are justified in light of the work of Perdew and Levy [33], where they commented on the significance of the highest occupied Kohn–Sham eigenvalue, and proved the ionization potential theorems for the exact Kohn–Sham density functional theory of a many–electron system. In addition the use of the energies of frontier molecular orbitals as an approximation to obtain *I* and *A* is supported by the Janak’s Theorem [34]. In particular, The negative of Hartree–Fock and Kohn–Sham HOMO orbital has been found to define upper and lower limits, respectively, for the experimental values of the first ionization potential [35] thus validating the use of energies of Kohn–Sham frontier molecular orbital to calculate reactivity descriptors coming from Conceptual DFT.

The electrophilicity index *ω* represents the stabilization energy of the systems when it gets saturated by electrons coming from the surrounding:

(8) $$\omega =\frac{\mu^{2}}{2\eta }\approx \frac{{\left(I+A\right)}^{2}}{4(I-A)}\approx \frac{{\left(\varepsilon_{L}+\varepsilon_{H}\right)}^{2}}{4(\varepsilon_{L}-\varepsilon_{H})}$$

The electron donating (*ω*^−^) and electron accepting (*ω*^+^) powers have been defined as [36]:

(9) $$\omega^{-}=\frac{{\left(3I+A\right)}^{2}}{16(I-A)}$$

and

(10) $$\omega^{+}=\frac{{\left(I+3A\right)}^{2}}{16(I-A)}$$

It follows that a larger *ω*^+^ value corresponds to a better capability of accepting charge, whereas a smaller value of *ω*^−^ value of a system makes it a better electron donor. In order to compare *ω*^+^ with - *ω*^−^, the following definition of net electrophilicity has been proposed [37]:

(11) $$\Delta \omega^{\pm }=\omega^{+}-(-\omega^{-})=\omega^{+}+\omega^{-}$$

that is, the electron accepting power relative to the electron donating power.

## Results and discussion

The molecular structure of Naringenin was pre-optimized by starting with the readily available PDB structure, and finding the most stable conformer by means of the Conformers module of Materials Studio through a random sampling with molecular mechanics techniques and a consideration of all the torsional angles. The structure of the resulting conformer was then optimized with the M06, M06L, M06-2X and M06-HF density functionals in conjunction with the MIDIY basis set.

The validity of the Koopmans’ theorem within the DFT approximation is controversial. However, it has been shown [35] that although the KS orbitals may differ in shape and energy from the HF orbitals, the combination of them produces Conceptual DFT reactivity descriptors that correlate quite well with the reactivity descriptors obtained through Hartree-Fock calculations. Thus, it is worth to calculate the electronegativity, global hardness and global electrophilicity for the studied systems using both approximations in order to verify the quality of the procedures.

The HOMO and LUMO orbital energies (in eV), ionization potentials I and electron affinities A (in eV), and global electronegativity *χ*, total hardness *η*, and global electrophilicity *ω* of the Naringenin molecule calculated with the M06, M06L, M06-2X and M06-HF density functionals and the MIDIY basis set are presented in Table 1. The upper part of the table shows the results derived assuming the validity of Koopmans’ theorem and the lower part shows the results derived from the calculated vertical I and A. As can be seen from Table 1, the Koopman’s theorem holds approximately for the density functionals which include some percentage of HF exchange, but it fails in part for the M06L density functional (without inclusion of HF exchange) (for the electronegativiy and total hardness but not for the global electrophilicity).

**Table 1:** **HOMO and LUMO orbital energies (in eV), ionization potentials I and electron affinities A (in eV), and global electronegativity *χ *, total hardness *η *, and global electrophilicity *ω * of Naringenin calculated with the M06, M06L, M06-2X and M06-HF density functionals and the MIDIY basis set**

**Property**	**M06**	**M06L**	**M06-2X**	**M06-HF**
HOMO	-5.9155	-4.7242	-7.3281	-9.2653
LUMO	-0.6052	-1.4060	0.1298	1.4321
*χ*	3.2604	3.0651	3.5992	3.9166
*η*	2.6552	1.6591	3.7290	5.3487
*ω*	2.0018	2.8313	1.7370	1.4339
I	7.4000	6.9619	8.1214	8.8799
A	0.9393	0.7328	0.9528	0.8997
*χ*	4.1697	3.8474	4.5371	4.8898
*η*	3.2304	3.1146	3.5843	3.9901
*ω*	2.6911	2.3763	2.8716	2.9962

The upper part of the table shows the results derived assuming the validity of Koopmans’ theorem and the lower part shows the results derived from the calculated vertical I and A.

The condensed Fukui functions can also be employed to determine the reactivity of each atom in the molecule. The corresponding condensed functions are given by $f_{k}^{+}=q_{k}(N+1)-q_{k}(N)$ (for nucleophilic attack), $f_{k}^{-}=q_{k}(N)-q_{k}(N-1)$ (for electrophilic attack), and $f_{k}^{0}=[q_{k}(N+1)-q_{k}(N-1)]/2$ (for radical attack), where *q*_*k*_ is the gross charge of atom *k* in the molecule.

It is possible to evaluate condensed Fukui functions from single-points calculations directly, without resorting to additional calculations involving the systems with N-1 and N+1 electrons:

(12) $${f_{k}}^{+}=\underset{a\in k}{\sum }\left[{c_{\text{ai}}}^{2}+c_{\text{ai}}\underset{b\neq a}{\sum }c_{\text{bi}}S_{\text{ab}}\right]\;\;\;\;\text{(where i = LUMO)}$$

and

(13) $${f_{k}}^{-}=\underset{a\in k}{\sum }\left[{c_{\text{ai}}}^{2}+c_{\text{ai}}\underset{b\neq a}{\sum }c_{\text{bi}}S_{\text{ab}}\right]\;\;\;\;\text{(where i = HOMO)}$$

with c_*ai*_ being the LCAO coefficients and S_*ab*_ the overlap matrix. The condensed Fukui functions are normalized, thus $\underset{k}{\sum }f_{k}=1$ and $f_{k}^{0}=[f_{k}^{+}+f_{k}^{-}]/2$.

The condensed Fukui functions have been calculated using the AOMix molecular analysis program [38,39] starting from single-point energy calculations. We have presented, discussed and successfully applied the described procedure in our previous studies on different molecular systems [40-43].

The condensed dual descriptor has been defined as $f^{\left(2\right)}{\left(r\right)}_{k}=f_{k}^{+}-f_{k}^{-}$[5,9]. From the interpretation given to the Fukui function, one can note that the sign of the dual descriptor is very important to characterize the reactivity of a site within a molecule toward a nucleophilic or an electrophilic attack. That is, if *f*^(2)^(**r**)_*k*_ > 0, then the site is favored for a nucleophilic attack, whereas if *f*^(2)^(**r**)_*k*_ < 0, then the site may be favored for an electrophilic attack [5,9,44].

The electrophilic f^−^ and nucleophilic f^+^ condensed Fukui functions and *f*^(2)^(**r**) over the atoms of the Naringenin molecule calculated with the M06, M06L, M06-2X and M06-HF density functionals and the MIDIY basis set are shown in Table 2. The actual values have been multiplied by 100 for an easier comparison.

**Table 2:** **Electrophilic f**^**−**^** and nucleophilic f**^**+**^** condensed Fukui functions and** ***f***^**(2)**^(**r**)** over the atoms of the Naringenin molecule calculated with the M06, M06L, M06-2X and M06-HF density functionals and the MIDIY basis set**

	**M06**			**M06L**			**M06-2X**			**M06-HF**		
**Atom**	**f**^**−**^	**f**^**−**^	***f***^**(2)**^**(*r *)**	**f**^**+**^	**f**^**−**^	***f***^**(2)**^**(*r *)**	**f**^**+**^	**f**^**−**^	***f***^**(2)**^**(*r *)**	**f**^**+**^	**f**^**−**^	***f***^**(2)**^**(*r *)**
1 O	2.13	0.99	1.14	2.32	0.35	1.97	1.58	1.23	0.35	1.05	0.50	0.55
2 C	11.39	0.30	11.09	10.86	0.29	10.57	11.68	0.28	11.48	11.58	0.06	11.52
3 C	1.36	1.26	0.10	1.27	1.21	0.06	1.45	0.99	0.46	1.62	0.88	0.74
4 C	0.61	0.73	-0.12	0.51	0.32	0.19	0.82	1.14	-0.32	1.38	0.23	1.15
5 C	6.30	6.30	0.00	5.23	8.06	-2.83	8.31	0.91	7.40	11.67	0.27	11.40
6 C	0.36	9.08	-8.72	0.30	10.14	-9.84	0.34	3.45	-3.11	0.33	2.46	-2.13
7 C	0.49	8.61	-8.12	0.41	2.57	-2.16	0.51	2.67	-2.16	0.63	2.75	-2.12
9 C	17.38	-0.01	17.39	16.22	-0.01	16.23	18.71	0.03	18.68	16.60	0.03	16.57
10 C	21.29	1.27	**20.02**	22.36	1.07	**21.29**	19.76	0.87	**18.89**	20.73	0.78	**19.95**
11 C	9.32	0.21	9.11	9.57	0.15	9.42	9.10	0.31	8.79	9.04	0.09	8.95
14 C	0.05	2.43	-2.38	0.07	1.05	-1.08	0.06	5.41	-5.35	0.07	6.81	-6.74
15 C	0.59	1.70	-1.11	0.42	0.81	-0.39	0.55	3.29	-2.74	0.64	4.18	-3.54
16 O	4.11	0.02	4.09	4.44	0.03	4.41	3.45	0.02	3.43	2.68	0.01	2.67
17 C	1.02	1.03	-0.01	0.69	0.54	0.15	1.53	0.84	0.69	2.24	0.11	2.13
18 O	18.58	44.09	**-25.51**	20.18	65.26	**-45.08**	17.95	45.56	**-27.61**	16.24	41.45	**-25.21**
19 O	2.31	0.98	1.33	2.65	1.10	1.55	1.81	0.40	1.41	1.28	0.06	1.22
20 C	0.20	3.63	-3.43	0.10	0.90	-0.80	0.19	9.54	-9.35	0.26	10.09	-9.83
23 C	0.05	3.72	-3.67	0.02	0.91	-0.89	0.03	10.56	-10.53	0.03	10.99	-10.96
28 C	0.32	5.50	-5.18	0.26	1.73	-1.47	0.34	15.69	-15.35	0.42	20.75	-20.33
30 O	0.08	7.15	-7.07	0.08	2.19	-2.11	0.06	15.62	-15.56	0.05	12.24	-12.19

The actual values have been multiplied by 100 for an easier comparison. H atoms are not shown.

It can be concluded from the analysis of the results on Table 2 that the M06, M06L, M06-2X and M06-HF density functionals predict that C10 will be the preferred site for nucleophilic attack. The four density functionals considered in this study display a large negative value of the condensed dual descriptor *f*^(2)^(**r**) over O18, implying that this will be the preferred site for the electrophilic attack.

The electrodonating (*ω*^−^) and electroaccepting (*ω*^+^) powers and net electrophilicity Δ*ω*^±^ of the Naringenin molecule calculated with the M06, M06L, M06-2X and M06-HF density functionals and the MIDIY basis set are presented in Table 3. The upper part of the table shows the results derived assuming the validity of Koopmans’ theorem and the lower part shows the results derived from the calculated vertical I and A.

**Table 3:** **Electrodonating (*ω***^**−**^**) and electroaccepting (*ω***^**+**^**) powers and net electrophilicity *Δ ω***^**±**^** of Naringenin calculated with the M06, M06L, M06-2X and M06-HF density functionals and the MIDIY basis set**

**Property**	**M06**	**M06L**	**M06-2X**	**M06-HF**
*ω*^−^	3.9637	4.5712	4.0026	4.0609
*ω*^+^	0.7035	1.5061	0.4035	0.1443
Δ*ω*^±^	4.6672	6.0773	4.4061	4.2052
*ω*^−^	6.2176	4.6892	5.5882	5.9398
*ω*^+^	1.2124	0.8419	1.0511	1.0500
Δ*ω*^±^	7.4300	5.5311	6.6393	6.9898

The upper part of the table shows the results derived assuming the validity of Koopmans’ theorem and the lower part shows the results derived from the calculated vertical I and A.

The results from Table 3 clearly indicate that Naringenin is an electrodonating molecule, with the same result predicted by all the four density functionals considered in this study. However, although the tendency is the same, the results for these descriptors are in poor agreement between those calculated assuming the validity of the Koopmans’ theorem, and those coming from energy differences.

## Conclusions

From the whole of the results presented in this contribution it has been clearly demonstrated that the sites of interaction of the Naringenin molecule can be predicted by using DFT-based reactivity descriptors such as the hardness, softness, and electrophilicity, as well as Fukui function calculations. These descriptors were used in the characterization and successfully description of the preferred reactive sites and provide a firm explanation for the reactivity of the Naringenin molecule.

The M06 family of density functionals (M06, M06L, M06-2X and M06-HF) used in the present work leads to the same qualitatively and quantitatively similar description of the chemistry and reactivity of the Naringenin molecule, yielding reasonable results. However, for the case of the M06-2X and M06-HF density functionals, which include a large portion of HF exchange, the calculations considering the validity of the Koopmans’ theorem lead to negative electron affinities.

The calculated descriptors are in agreement with the known experimental facts about the chemical reactivity of the Naringenin molecule presented in the literature (with the exceptions mentioned on the paragraph above). Thus, this make us confidents that similar studies can be pursued with the same degree of accuracy on another flavonoids with analogue structures.

## Competing interests

The author declares that he has no competing interests.
